# Supplementary material for: Inkjet-printed optical interference filters
Source: Nat Commun. 2024 Apr 20;15:3372. doi: 10.1038/s41467-024-47086-x (PMC11032308; doi:10.1038/s41467-024-47086-x)
Supplement: Supplementary file 1 — Supplementary information [file 41467_2024_47086_MOESM1_ESM.pdf]

Supplementary Information

Inkjet-printed Optical Interference Filters

Qihao Jin[1,*], Qiaoshuang Zhang[1], Christian Rainer[1,2], Hang Hu[1,3], Junchi Chen[1], Tim Gehring[1], Jan Dycke[1], Roja Singh[1,3], Ulrich. W. Paetzold[1,3], Gerardo Hernandez-Sosa[1,2,3], Rainer Kling[1], and Uli Lemmer[1,2,3,*]

[1]Light Technology Institute (LTI), Karlsruhe Institute of Technology (KIT), Engesserstrasse 13, 76131 Karlsruhe, Germany

[2]InnovationLab, Speyerer Strasse 4, 69115 Heidelberg, Germany

[3]Institute of Microstructure Technology (IMT), Karlsruhe Institute of Technology (KIT), Hermann-von-Helmholtz-Platz 1, 76344 Eggenstein-Leopoldshafen, Germany

*E-mail: qihao.jin@kit.edu; uli.lemmer@kit.edu

Contents

1. Comparison between optical interference filters and color filters ... 3
2. Ink design and formulation ... 4
3. Characterization of ink printability ... 7
4. Calculation of We_j in method 2 ... 8
5. Refractive index and extinction coefficient of printed layers ... 10
6. Nozzle clogging investigation on inks with different organic vehicles ... 11
7. Thin film homogeneity ... 12
8. Thickness control of inkjet-printed layer ... 15
9. Surface roughness of the printed layers ... 16
10. Reproducibility of the printing process and the printed filters ... 17
11. Comparison of ripples in the transmitting curve between periodic and non-periodic structures ... 19
12. Sites in 2.5 × 2.5 cm^2 bandpass filter for optical homogeneity characterization ... 20
13. 10 × 10 cm^2 dichroic filter ... 21
14. A4 size (29.7 × 21.0 cm^2) dichroic filter ... 23
15. Printing OIFs on flexible foil ... 24
16. Residual transmission elimination ... 25
17. Comparison with commercial products ... 26
18. Durability test of printed layers ... 29
19. Crystal structure characterization of nanoparticles ... 32
20. Designed thickness of the filters ... 33
21. Transmittance of glass substrate ... 36

The following table summarizes the key information of the sections

Section 1:	Highlighting the difference between “multilayer interference filter” and “color filters”
Section 2-4:	Ink development related
Section 5:	Refractive index of the materials
Section 6-9:	Printing process optimization and layer quality related
Section 10-15:	Properties of inkjet-printed optical interference filters
Section 16-17:	Performance enhancement and comparison with commercial products
Section 18:	Durability test according to standards
Section 19:	Crystal structures of nanoparticles
Section 20:	Designed thickness values of layers
Section 21:	Transmittance curve of the glass substrate

1. Comparison between optical interference filters and color filters

The novelty of this work is the successful inkjet printing of optical interference filters (OIFs), which consist of a multilayer structure. In this section, we show an overview of the difference between the OIFs and color filters, which are based on absorptive materials such as dyes or pigments.

To better illustrate the differences between the OIFs and the color filters, some major properties are included in Supplementary Table 1 for reference.

Supplementary Table 1. Differences between OIFs and color filters[1–11].

	Dielectric interference filters	Color filters
Physical principle	Optical interference	Absorption
Differences in fabrication		
Layer structure	Multi thin-layer stacks	One thick layer
Typical single-layer thickness	~10-300 nm	In the micrometer to millimeter range
Layer thickness control	Elaborate (on nm scale)	Rough (on sub-μm scale)
Fabrication complexity	High	Low
Differences in the filter's property		
Energy loss	Nearly zero	Severe
Heat stability	High	Medium to Low
Light stability	High	Medium to Low
Spectral profile	High degree of freedom based on design	Limited by absorbing materials
Spectral precision	Good to <1 nm	Limited by absorbing materials, >10 nm
Spectral bandwidth (FWHM)	Narrow to <1 nm	Limited by absorbing materials, >100 nm
Laser application suitability	High	Low
Example of a transmission spectrum	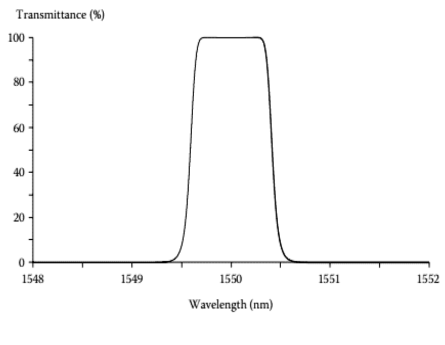	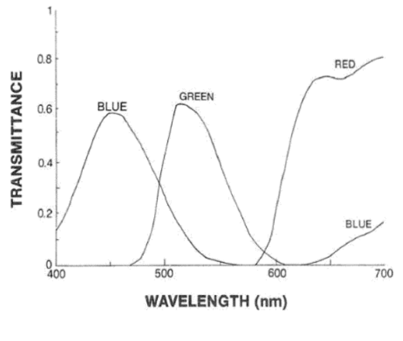

2. Ink design and formulation

The inks formulated in our work were based on SiO_2 and TiO_2 nanoparticle dispersions. In inkjet printing, ink droplets are ejected from nozzles and merged to form a thin film after being printed on a surface. This process is dominated by a number of physical parameters. The crucial ones are the surface free energy (SFE) of the solid layer on which the ink is printed, surface tension (SFT), viscosity, and vapor pressure of the ink. One of the main tasks of ink formulation in this work was to develop inks that show a low contact angle (CA) on the solid surface. Moreover, to simplify the fabrication process, pre-surface-treatment of the solid layer using UV/Ozone plasma or other additional steps were preferred to be excluded as this would lead to a complex deposition procedure. To obtain a low CA, a proper match between the SFE of the layer and the SFT of the liquid is required. Starting from the relation among liquid, solid surface, and vapor, Young proposed the equation[12]

$$\gamma_{SV} = \gamma_{SL} + \gamma_{LV} cos\theta \tag{1}$$

where γ_{SV}, γ_{SL}, and γ_{LV} are the interfacial tension of solid-vapor, solid-liquid, and liquid-vapor, respectively. θ denotes the CA. Furthermore, γ_{LV} can be considered as the sum of two sub-tensions[13], i.e.

$$\gamma_{LV} = \gamma_L^D + \gamma_L^P \tag{2}$$

where D and P refer to the force caused by the dispersive and polar components, respectively. The relation between θ and SFE of the solid surface, therefore, can be expressed as[13]:

$$cos\theta = 2\frac{\sqrt{\gamma_S^D \gamma_L^D} + \sqrt{\gamma_S^P \gamma_L^P}}{\gamma_{LV}} - 1 \tag{3}$$

Using knwon γ_L^D and γ_L^P values of two reference liquids, γ_S^D, γ_S^P can be calculated from measured CAs. And the total SFE equals the sum of γ_S^D and γ_S^P. In this work, the OIFs were fabricated using SiO_2 and TiO_2 inks, which were alternately printed on solidified surfaces. Therefore, determining the SFE of SiO_2 and TiO_2 surfaces was essential for a proper ink design. Here, three reference liquids were used to determine the SFE of surfaces, i.e., deionized water (H_2O), ethylene glycol (EG), and diiodomethane (DIM). CAs were measured on ten different sites on each surface with each reference liquid (Supplementary Fig. 1a), where the black bars represent averaged values. The average SFE of the SiO_2 layer was measured as 68.24 mN m^{-1} with the dispersive share of 32.55 mN m^{-1} and the polar share of 35.69 mN m^{-1}. The average SFE of the TiO_2 layer was measured as 66.16 mN m^{-1} with the dispersive share of 33.59 mN m^{-1} and the polar share of 32.57 mN m^{-1}. After knowing the SFE, it is possible to plot the wetting envelope of each surface (Supplementary Fig. 1b). The x- and y-axis represent the dispersive and polar share of the SFT of a liquid, respectively. Here, three wetting envelopes (CA=0°) of different surfaces are plotted, belonging to the SiO_2 surface (blue), the TiO_2 surface (salmon), and the glass substrate (yellow), respectively. When the SFT of a liquid locates within a certain envelope, it is considered a complete wetting of the liquid on this surface[14]. Therefore, by measuring the SFT and its components, the wettability of inks on the corresponding surface can be evaluated.

Ink formulation was done by dispensing oxide nanoparticles in selected organic vehicles. For the high RI ink, TiO_2 nanoparticles with a size of around 13 nm were dispersed in 2-propoxyethanol at a solid concentration of 1.8 wt%. For the low RI ink, SiO_2 nanoparticles with

a size of around 10 nm were first dispersed in methyl ethyl ketone (MEK) at a concentration of 40 wt%. However, MEK has a low boiling point and is highly volatile. In large-scale production, it neither fulfills the requirements in volatile organic compounds legislation[15], nor is it a suitable solvent to avoid nozzle clogging[16]. Therefore, 1,3-dimethoxybenzene was applied to replace MEK (see Methods for details) as the eventual organic vehicle of SiO_2 dispersion (solid concentration of 3 wt%). This organic vehicle exchange helped establish a stable printing process (e.g., low nozzle clogging risk without the need for humectants) and homogenous layer formation, which will be discussed in the following. The SFT of each ink was measured. The details of determining the total SFT and its dispersive and polar parts are presented in the Methods. The droplet profiles used for the total SFT determination of each ink are shown (Supplementary Fig. 1c I and II). The SFT of SiO_2 ink was measured as 36.84 mN m^{-1}, with the dispersive part of 36.58 mN m^{-1} and the polar part of 0.26 mN m^{-1}, respectively. The average SFT of TiO_2 ink was measured as 27.70 mN m^{-1} with the dispersive part of 25.17 mN m^{-1} and the polar part of 2.53 mN m^{-1}, respectively. The SFTs are then plotted in the wetting envelope diagram to evaluate ink wettability on solid surfaces (Supplementary Fig. 1b). It can be seen that the SFT values of the TiO_2 and SiO_2 inks are located within their corresponding envelopes. Both nanoparticle inks show good wettability on the counterpart surface, i.e., SiO_2 on TiO_2 and vice versa.

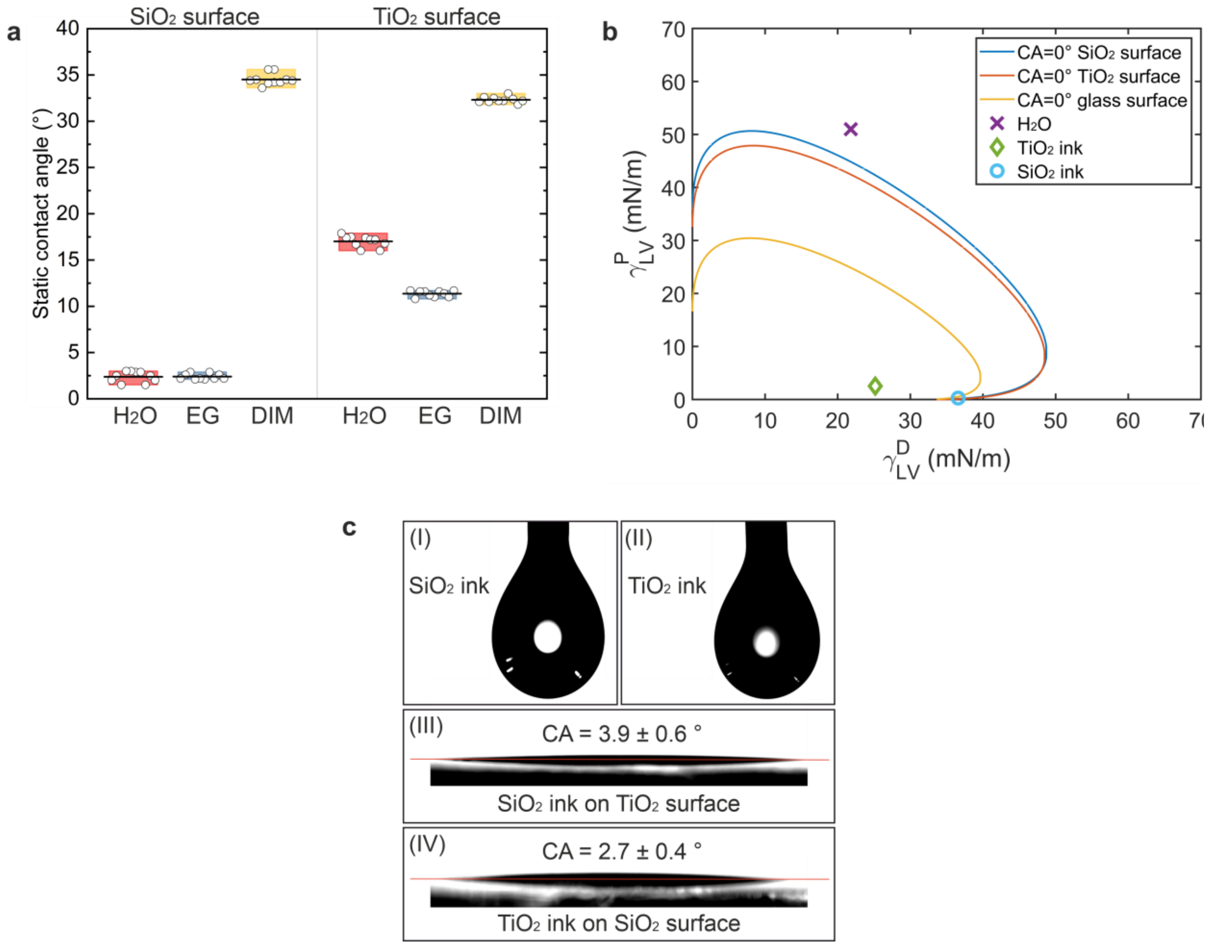

Supplementary Fig. 1. Ink design and formulation. (a) Measured contact angles for surface free energy characterization. (b) Wetting envelope diagram. (c) Ink surface tension and contact angle measurements.

In this work, the main reasons for formulating 1,3-dimethoxybenzene- and 2-propoxyethanol-based dispersions are the following: First, both dispersions showed excellent wettability on corresponding surfaces. The CA of the respective ink on the corresponding surface was 3.9 ± 0.6 ° for the SiO_2 ink on the TiO_2 surface, and 2.7 ± 0.4 ° for the TiO_2 ink on the SiO_2 surface (Supplementary Fig. 1c III and IV). These low CAs verified the results from the wetting envelope diagram. Second, both dispersions showed less nozzle clogging when compared to those based on other more common organic vehicles (Supplementary Section 5). Third, the ink formulations were humectant-free. The thin film layer homogeneity under the existence of humectants in inks was investigated (Supplementary Section 6). In practice, inks used for inkjet printing mostly contain humectants to prevent nozzle clogging. Although a common strategy to exclude the humectant is to use a low-solid-concentration ink, it requires either ultrahigh printing resolution or multi-printing cycles to reach the desired single-layer thickness. Both additional technical efforts are not suitable for industrial manufacturing. The developed inks in this work not only achieved a proper concentration to fulfill a layer deposition in one printing cycle, but also managed to form an excellent thin film homogeneity and maintain an ideal nozzle printing state.

3. Characterization of ink printability

In the characterization of the ink printability, two methods were used. Besides the method one described already in the main text, a second method (Supplementary Fig. 2) is also applied in this work to complement the evaluation of the ink printability[17]. It is proposed that to assess the mechanism during the ink ejection phase more precisely, *We* can be replaced by We_j, which is based on the fluid velocity inside the nozzle, v_j, prior to the droplet formation. The *Re*-*We* space diagram is thus mapped to a *Z*-We_j space diagram to present the ink usability more fairly, as *Z* contains only the physical properties of the ink itself. The detailed calculation of We_j is shown in Supplementary Section 3. Depending on sufficient previous empirical work, a printable region can also be predicted. The printable criterion We_j is defined as between 2 and 25[17]. In this diagram, the boundary conditions are briefly concluded as droplet generation threshold (red line) and single-satellite droplet formation (black line). Below the red line, the liquid has insufficient energy to overcome the capillary force to be ejected; and above the black line, the ejected droplets form extended tails. Lines of the developed ink in this work are plotted in the *Z*-We_j space diagram. The location of both ink lines implies the suitability for inkjet printing, in agreement with our findings above. For clarity, the rheological properties of developed inks are summarized (Supplementary Table 2).

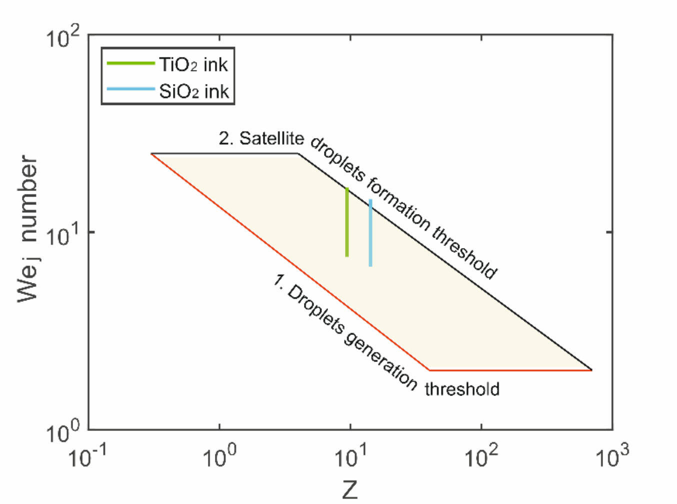

Supplementary Fig. 2. Characterization of ink printability using method 2.

Supplementary Table 2. Properties of developed inks.

	SiO_2 ink	TiO_2 ink
Nanoparticle concentration	3.0%	1.8%
Surface tension (mN m^{-1})	36.84<br>(Dispersive: 36.58; Polar: 0.26)	27.70<br>(Dispersive: 25.17; Polar: 2.53)
Density (g ml^{-1})	1.069	0.934
Viscosity (mPa·s)	2.04	2.5
Droplet velocity (m s^{-1})	2-4	2-4
Re number	22.53-45.07	16.05-32.10
We number	2.50-9.98	2.90-11.60
Oh number	0.07	0.11
Z number	14.26	9.43

4. Calculation of We_j in method 2

In method 2, We_j was used in the printability evaluation. We_j depends on v_j, which is the fluid velocity inside the nozzle, prior to the droplet formation.

$$We_j = \frac{v_j^2 \rho d}{\gamma} \quad (1)$$

where d is the characteristic length (in m, which is the nozzle orifice size). γ, η, and ρ are the surface tension (in N m^{-1}), dynamic viscosity (in Pa s), and density (in kg m^{-3}) of the ink, respectively. Since the volume of the ejected droplet, V_j, can be calculated as the integral of the fluid flow rate through the nozzle, assuming the nozzle has an exit area of A[18],

$$V_j = \int_{t_0}^{t_e} Av dt \quad (2)$$

where v is the ejected droplet velocity (in m s^{-1}). The fluid inside the nozzle starts to flow due to the meniscus at t_0, and the droplet is ejected from the nozzle at t_e with a velocity v smaller than v_j.

Reduced momentum of fluid advection is partially attributed to the viscus dissipation in the process of pinching off. The transferred momentum, p_v, due to viscous friction can be calculated as

$$p_v = -3\eta A \quad (3)$$

where η is the dynamic viscosity. The surface tension is another factor that reduces the droplet momentum, attributed to capillary force. The transferred momentum, p_c, due to capillary force can be calculated as

$$p_c = (t_e - t_0)\pi r_n \gamma \quad (4)$$

where γ is the surface tension, and r_n is the nozzle radius, which equals the ink droplet radius when the latter is ejected from the nozzle. Combining the equation (2), (3), and (4), the ejected droplet velocity v can be estimated as

$$v = \frac{1}{\rho V_j}\left(\int_{t_0}^{t_e} \rho A v_j^2 dt - 3\eta A - (t_e - t_0)\pi r_n \gamma\right) \quad (5)$$

Taking the nozzle as a circular section,

$$A = \pi r_n^2 \quad (6)$$

consider the time interval $\Delta t = t_e - t_0$. Therefore, V_j equals to $\pi r_n^2 v_j \Delta t$; finally, the equation (5) can be written as[17]

$$v_j^2 - v_j v - \frac{3\eta}{\rho \Delta t} - \frac{\gamma}{\rho r_n} = 0 \quad (7)$$

Hence, by a known Δt, v_j can be calculated by a known v. According to equation (2),

$$V_j = \pi r_n^2 v_j \Delta t \quad (8)$$

Combining equations (7) and (8), V_j can be rewritten as

$$V_j = \pi {r_n}^2 \frac{v + \sqrt{v^2 - 4 \times (-\frac{3\eta}{\rho \Delta t} - \frac{\gamma}{\rho r_n})}}{2} \Delta t \tag{9}$$

According to the droplet analysis system of the printer, the V_j and v are known, which are 10 pL and 4 m/s, respectively. Therefore, Δt is determined as 6 µs. Furthermore, We_j can be calculated accordingly by using equation (1).

5. Refractive index and extinction coefficient of printed layers

The refractive indices of the solid layers were measured within the wavelength range of 400 to 800 nm (Supplementary Fig. 3). The RI of the SiO_2 layer remains almost constant throughout the entire spectral region, ranging from 1.39 at 400 nm to 1.36 at 800 nm. The RI of the TiO_2 layer exhibits a slight decrease from 2.04 at 400 nm to 1.83 at 800 nm. However, the overall spectral RI contrast is high enough for fabricating the OIFs. On the other hand, within the measured wavelength range, the SiO_2 layer exhibits an almost negligible extinction coefficient, indicating excellent optical performance with minimal loss. However, for the TiO_2 layer, there is a slight increase in the extinction coefficient observed for shorter wavelengths. From our previous work[19], this extinction can be suppressed to zero by adding a proper amount of polymer into the TiO_2 nanoparticle ink.

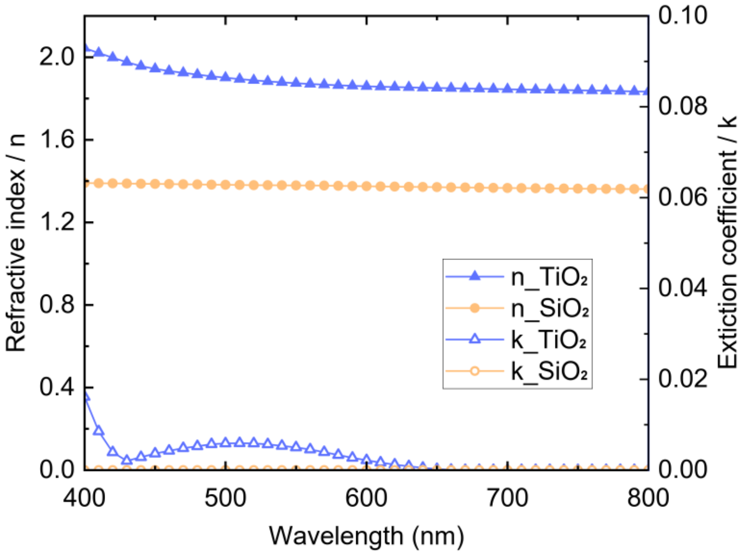

Supplementary Fig. 3. Refractive index and extinction coefficient of printed layers.

6. Nozzle clogging investigation on inks with different organic vehicles

SiO_2 dispersions

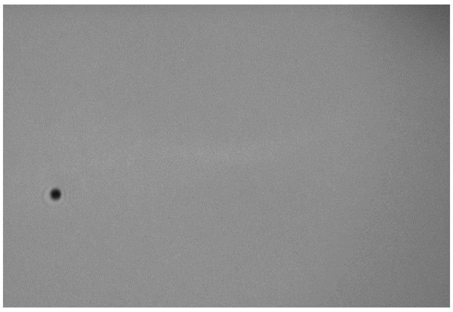

a MEK-SiO_2 dispersion (3%), 1 printing cycle

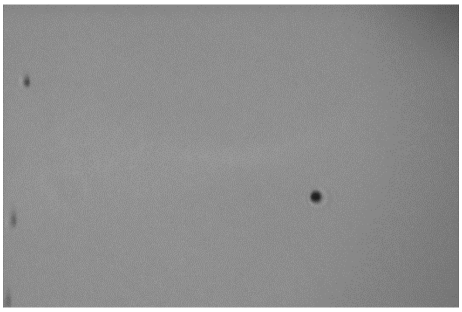

b IPA-SiO_2 dispersion (3%), 1 printing cycle

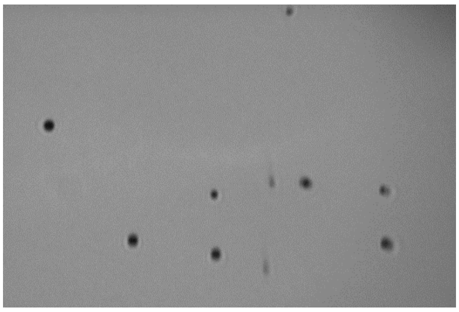

c Water-SiO_2 dispersion (3%), 1 printing cycle

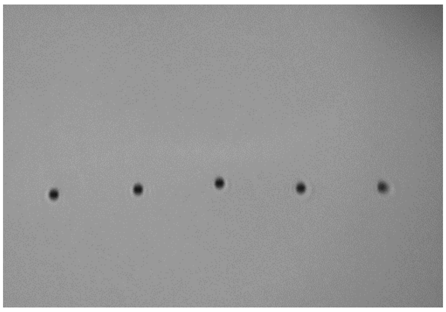

d Developed SiO_2 dispersion (3%), 5 printing cycles

TiO_2 dispersions

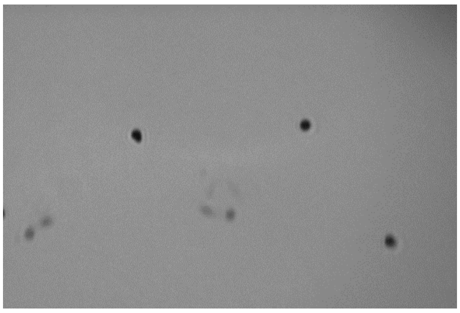

e Ethanol-TiO_2 dispersion (1.8%), 1 printing cycle

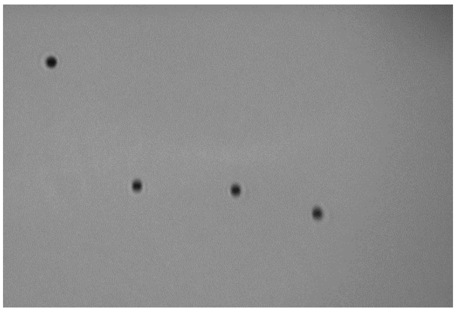

f IPA-TiO_2 dispersion (1.8%), 1 printing cycle

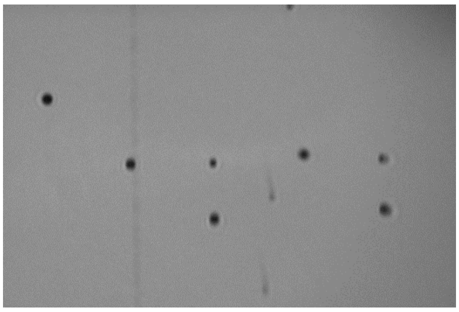

g Water-TiO_2 dispersion (1.8%), 1 printing cycle

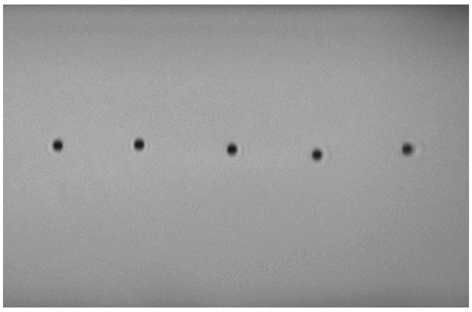

h Developed TiO_2 dispersion (1.8%), 5 printing cycles

Supplementary Fig. 4. Nozzle jetting status after certain printing cycles. **a-d** SiO_2 dispersions. **e-h** TiO_2 dispersions. **d** and **h** show nozzle jetting status after 5 printing cycles. The rest of the images show nozzle jetting status only after 1 printing cycle.

7. Thin film homogeneity

7.1. Humectant investigation

Including humectants in the inks is common for preventing the nozzles from clogging. 1,3-Propanediol is a common humectant in inks for inkjet printing. Printing with the developed humectant-free TiO_2 ink in this work showed no apparent nozzle clogging at nozzles over 5 printing cycles. However, it is worth investigating ink formulation with additional humectant to further reduce the maintenance effort for large printing cycles. Therefore, TiO_2 Inks with different humectant concentrations of 30%, 20%, and 10%, respectively, were developed. All the inks with humectant were nozzle-clogging-free after 20 printing cycles, showing improved jetting behavior. However, the thin film homogeneity was heavily impacted by the existence of the humectant. Tear-like drops were formed during printing, as can be seen in Supplementary Fig. 5a, b, and c. The size of tear-like drops increases with the concentration of the humectant.

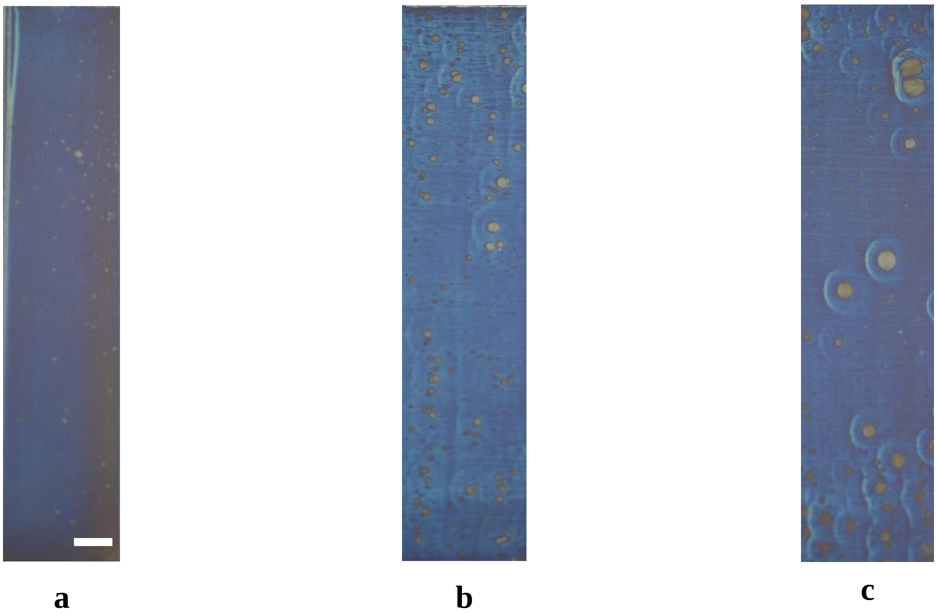

Supplementary Fig. 5. Printed TiO_2 thin films with different concentrations of 1,3-Propanediol as a humectant. **a** 30%. **b** 20%. **c** 10%. Scale bar 10 mm, applicable to all.

Moreover, the tear-like drops moved along a particular axis during printing, leaving a long tail in large-size printing, as seen in Supplementary Fig. 6a. The homogenous thin film was printed using developed humectant-free ink, as seen in Supplementary Fig. 6b. Considering the printing performance when using the formulated ink, including the very low nozzle clogging and homogenous thin film formation, the ink formulation then focused on humectant-free recipes.

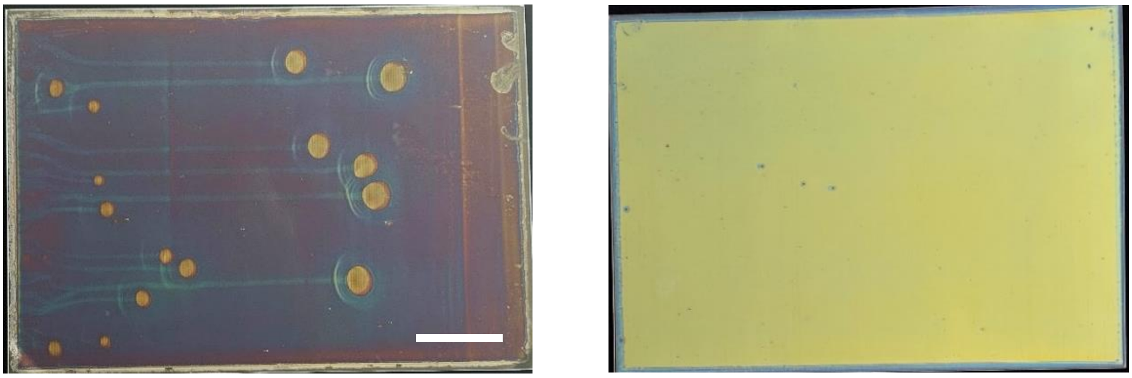

a b

Supplementary Fig. 6. Thin film quality investigation on printed A4 size filters with and without humectant. **a** Printing with inks using 1,3-Propanediol as a humectant. Concentration of humectant 10%. Tear-like drops on printed film. **b** Printing with inks without humectant. Scale bar 50 mm, applicable to both.

7.2. Substrate temperature optimization

During printing, the substrate temperature was set to 35, 30, and 25°C, respectively. The thin film homogeneity became better for the reduced substrate temperature.

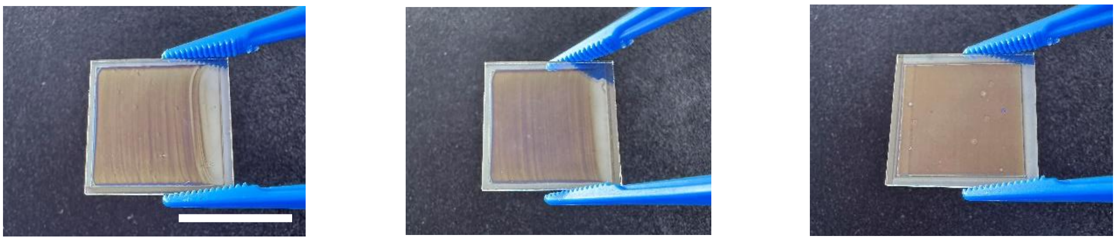

a b c

Supplementary Fig. 7. Substrate temperature impact on TiO_2 thin film homogeneity. **a** 35°C. **b** 30°C. **c** 25°C. The scale bar in **a** is 20 mm, applicable to all.

7.3. Printing speed optimization

When the drying rate of the printed wet line remains constant, the merge of former and subsequent lines is crucial for homogenous thin film formation[20]. In this work, to investigate the optimal printing speed, the speed value was set to 50 mm s^{-1}, 125 mm s^{-1}, 150 mm s^{-1}, and 200 mm s^{-1}, respectively. In the case of a relatively low printing speed, 50 mm s^{-1}, many ridge lines were found, which is attributed to the insufficient merge of wet ink lines during drying. In the case of relatively high printing speeds, 150 mm s^{-1} and 200 mm s^{-1}, no ridge lines were found, but large stripes existed. These stripes were probably caused by strong eddy flows within the gap between the moving printhead and substrate due to the high printing speed[21].

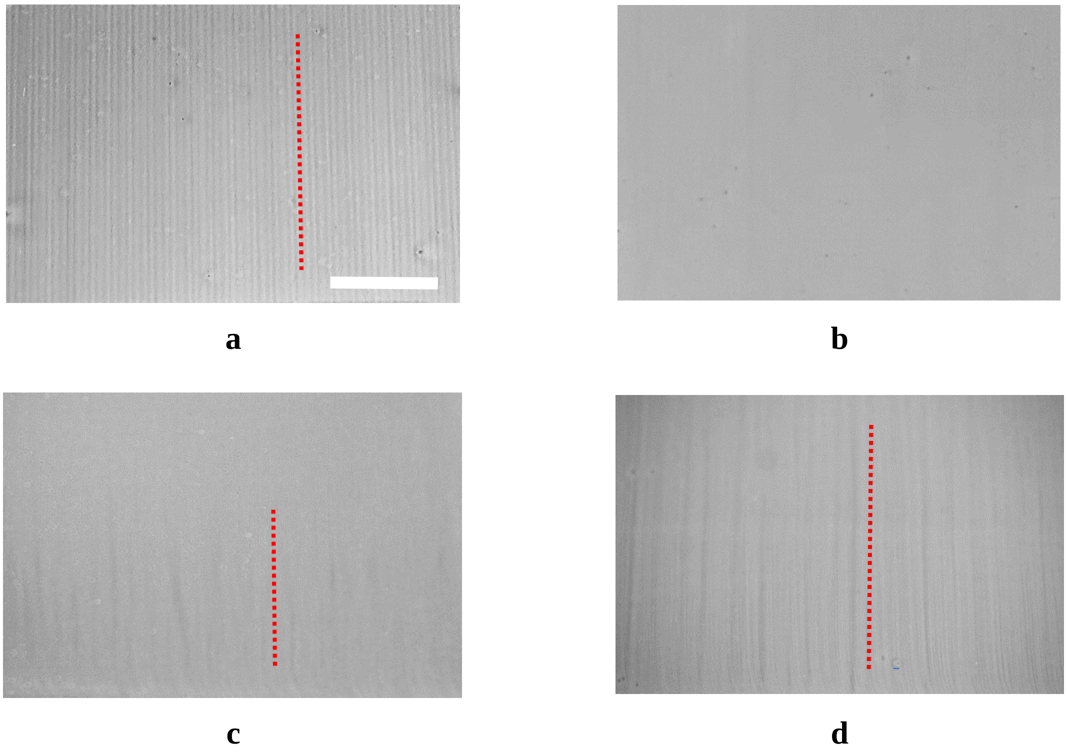

Supplementary Fig. 8. Printing speed impact on TiO_2 thin film homogeneity. **a** 50 mm s^{-1}. **b** 125 mm s^{-1}. **c** 150 mm s^{-1}. **d** 200 mm s^{-1}. Scale bar 5 mm, applicable to all. Red dashed lines indicate inhomogeneous defect lines.

8. Thickness control of inkjet-printed layer

The strategy of thickness control in this work is realized by controlling the total amount of ink volume deposited on a unit area. The total deposited ink volume is governed by the number of printed droplets, controlled by the printing resolution, i.e., dots per inch (dpi).

Assume the number of printed droplets on a unit area is N_0, resulting in a thickness of T_0. For printing a thicker layer with a thickness of T_1, a droplet number of N_1 is needed.

$$T_1 - T_0 = \Delta T$$
$$N_1 - N_0 = \Delta N$$

A well-coupled relation between ΔT and ΔN is essential to realize good thickness control.

In this work, using formulated SiO_2 ink, different ink droplet numbers N_i (400k, 500k, 600k, 700k, 800k, 900k, where 400k denotes 400000 and the same rule applies for other numbers) were used to print on individual substrates with the same printing area of 25 × 25 mm^2. Layer thickness was measured by a profilometer (Dektak, Bruker). Each thickness was determined as the average value of multiple sites along the measuring path (0.5 mm long). For each N_i, printing was performed three times to show the repeatability of the thickness, which gives a standard deviation of the thickness. The results are shown in Supplementary Fig. 9.

It can be found that the thickness increases linearly with the N. The coefficient of determination is 0.9999, indicating an excellent regression. Therefore, a well-controlled thickness by changing the ΔN can be successfully achieved. Furthermore, for each printed thickness, individual deviation stays within 1% of the layer thickness, implying a highly reproducible thickness and a high repeatability of the printing process and results.

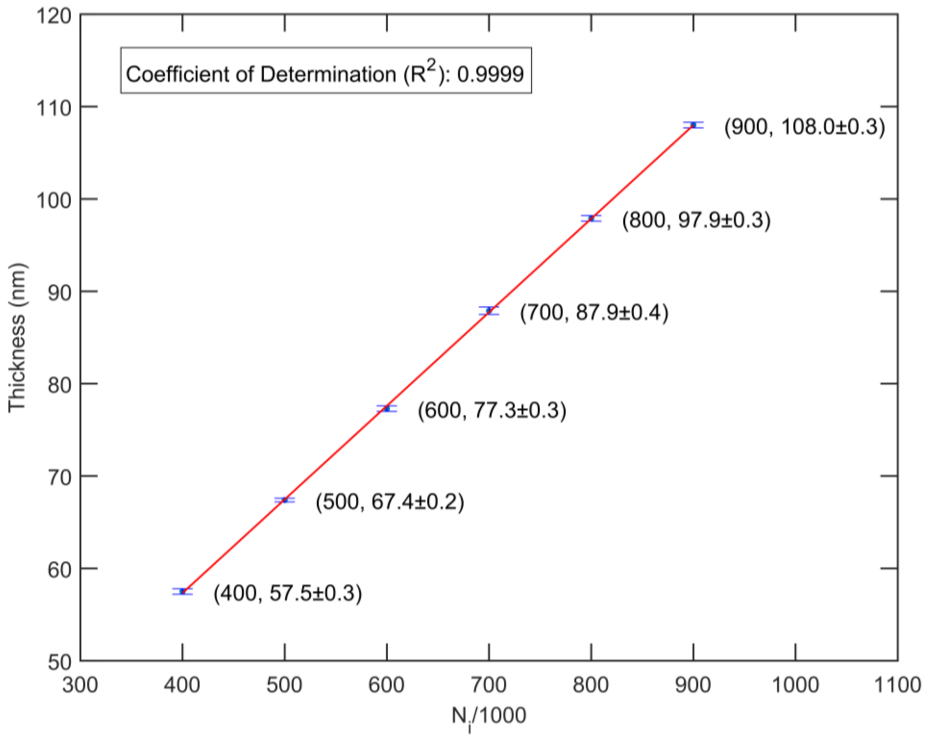

Supplementary Fig. 9. Thickness over the total amount of the printed droplet number N_i.

9. Surface roughness of the printed layers

Details of the roughness of the layers were investigated by atomic force microscopy (AFM).

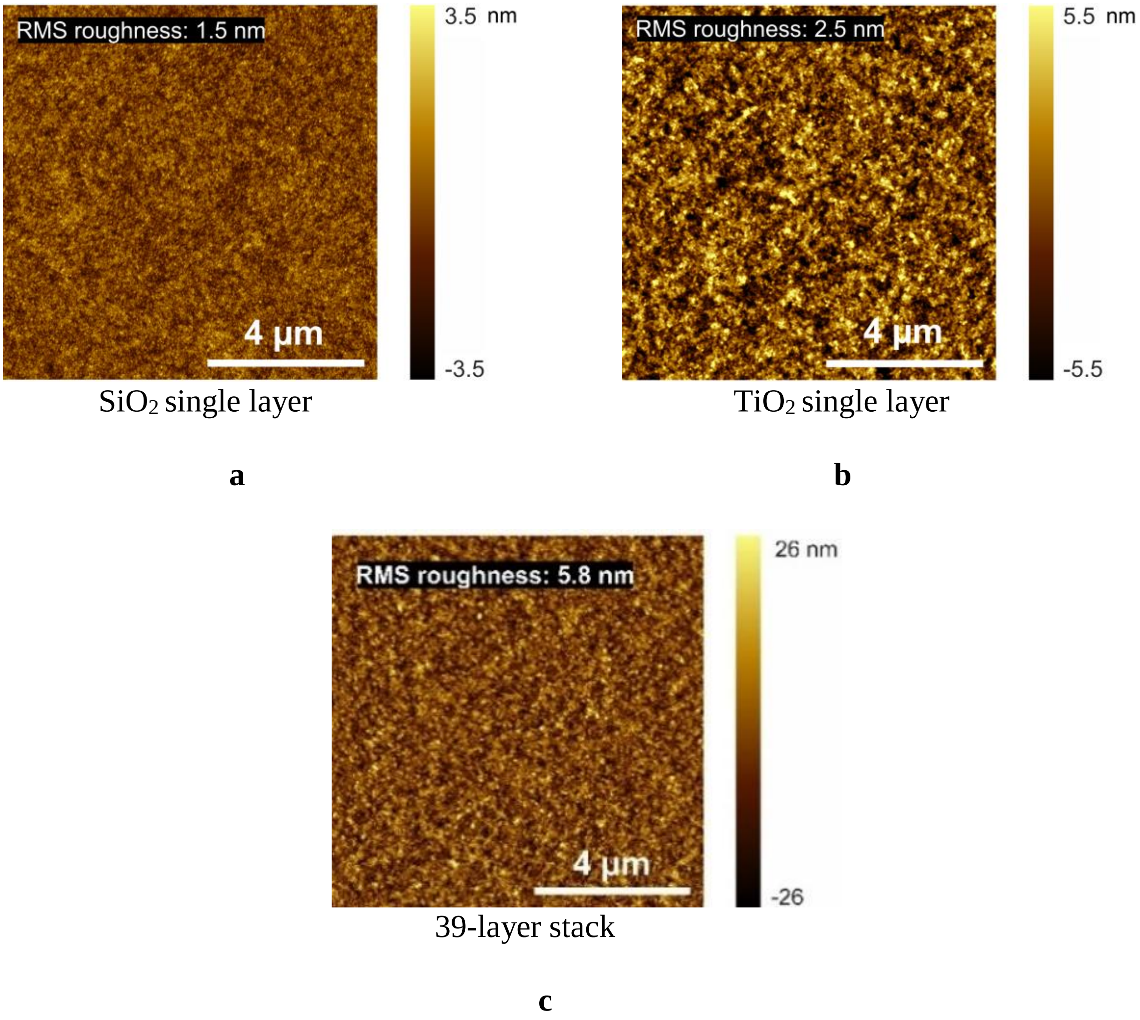

Supplementary Fig. 10. Surface roughness characterized by AFM. **a** Printed SiO2 Single layer. Thickness ≈ 100 nm. **b** Printed TiO2 Single layer. Thickness ≈ 100 nm. **c** Printed 39-layer stack. Thickness ≈ 3.3 µm.

10. Reproducibility of the printing process and the printed filters

To show the reproducibility of the printed filters and the printing process five dielectric filters with the same reflecting center wavelength were printed. The size of the filters is 25 × 25 mm^2, and the reflection spectra were measured in the center of the filters. The light spot used in the optical measurement is 5 mm in diameter. The measured spectra are shown in Supplementary Fig. 10, where the spectra in (a) are plotted separately and (b) are plotted in one.

It can be seen from the results that all five printed optical filters show only minor differences in the optical spectrum. The deviations of the wavelengths at the reflectance of 50% are ± 0.5 nm for the rising edge and ± 1 nm for the falling edge. These quantitative analysis indicate that the printing results are highly reproducible.

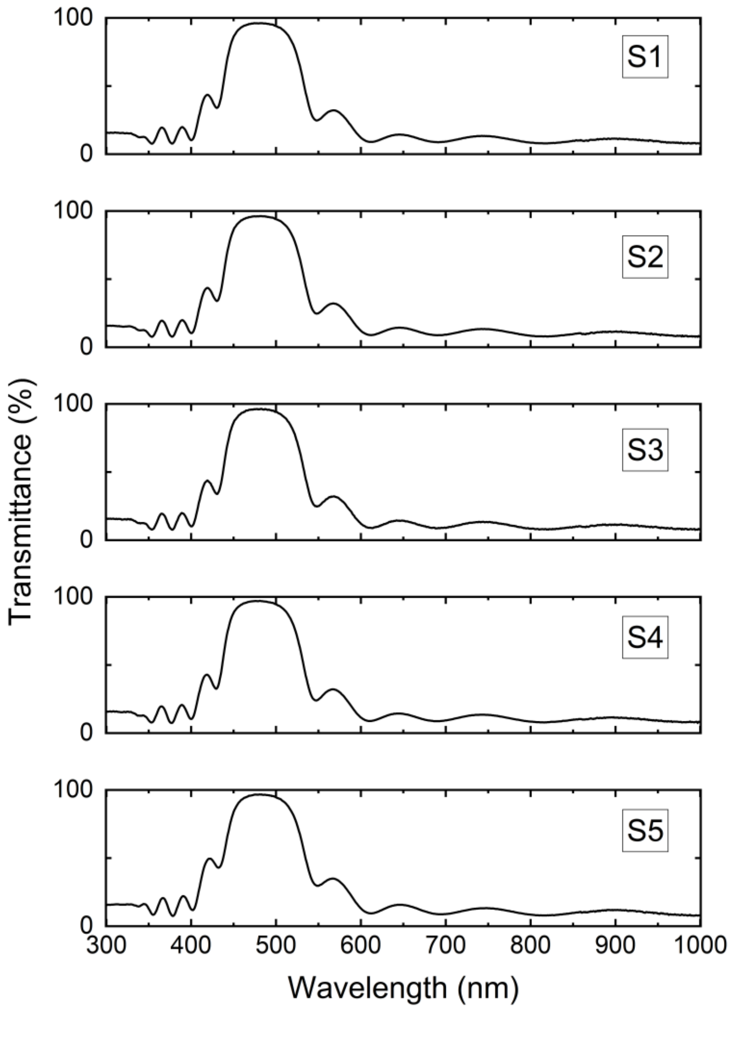

a

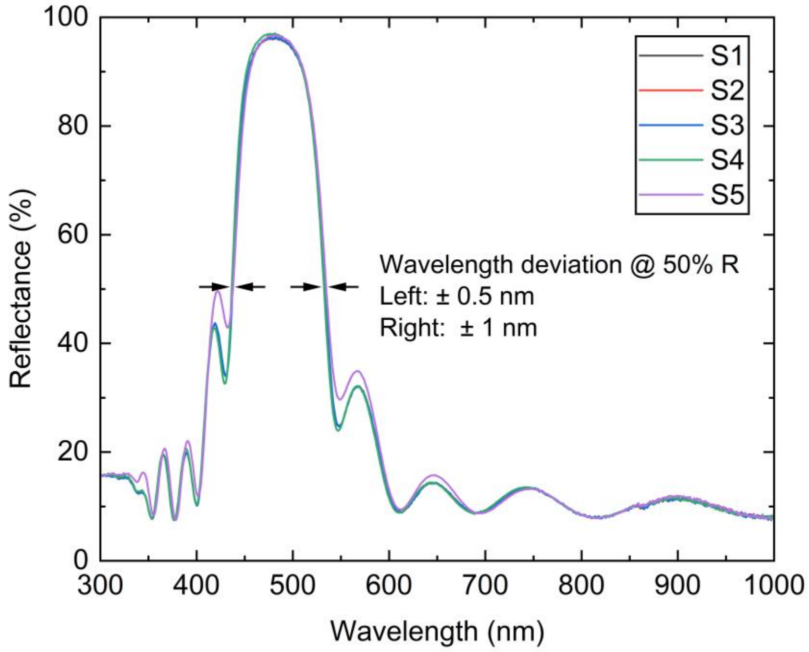

b

Supplementary Fig. 11. Measured reflection spectra of five printed filters. The wavelength deviations are measured at a refraction (R) of 50%. **a** Plotted separately. **b** Plotted in one.

11. Comparison of ripples in the transmitting curve between periodic and non-periodic structures

In this section, the transmission curves of the non-periodic and the periodic stack structures are compared. The non-periodic structure refers to the design in which individual layer thickness is optimized and used for inkjet printing. Therefore, the thickness of each layer can be different from others. Details can be found in Section 20.

As can be seen, the non-periodic structure with the optimized layer thickness results in lessened ripples in the transmission.

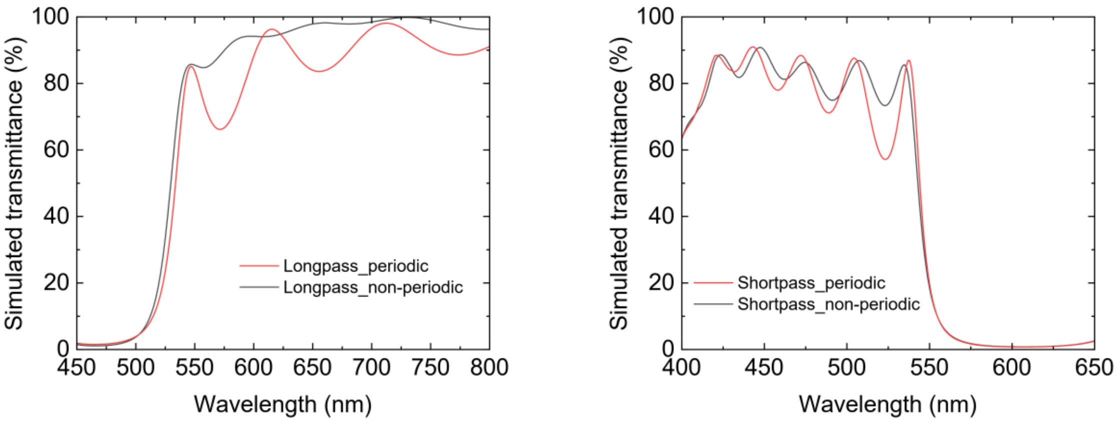

a **b**

Supplementary Fig. 12. Ripples lessening in non-periodic structures. Periodic structure as a comparison. **a** Longpass filter. **b** Shortpass filter.

12. Sites in 2.5 × 2.5 cm^2 bandpass filter for optical homogeneity characterization

To characterize the homogeneity of the optical property in the printed 2.5 × 2.5 cm^2 filter, an array consisting of 9 sites was used. Optical transmission measurements were performed at each site.

The size of the light spot was 2 mm. The distance between the neighboring sites is 3 mm.

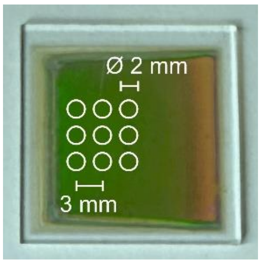

Supplementary Fig. 13. Sites for optical uniformity characterization in 2.5 × 2.5 cm^2 size filter.

13. 10 × 10 cm^2 dichroic filter

13.1. Normalized reflection spectrum

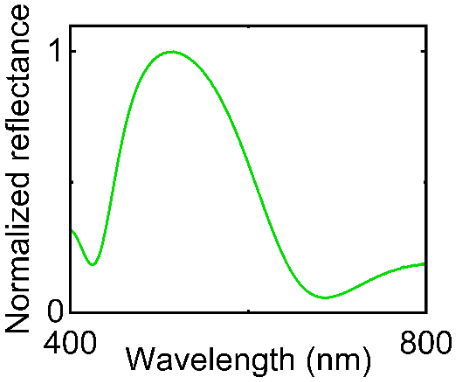

Supplementary Fig. 14. Normalized reflection spectrum of 10 × 10 cm^2 dichroic filter.

13.2. 20 sites for characterizing the homogeneity of optical property

To characterize the uniformity of the optical property in the printed 10 × 10 cm^2 filter, an array consisting of 20 sites was used. Optical transmission measurements were performed at each site. The size of the light spot was 2 mm. The distance between the neighboring site is 20 mm.

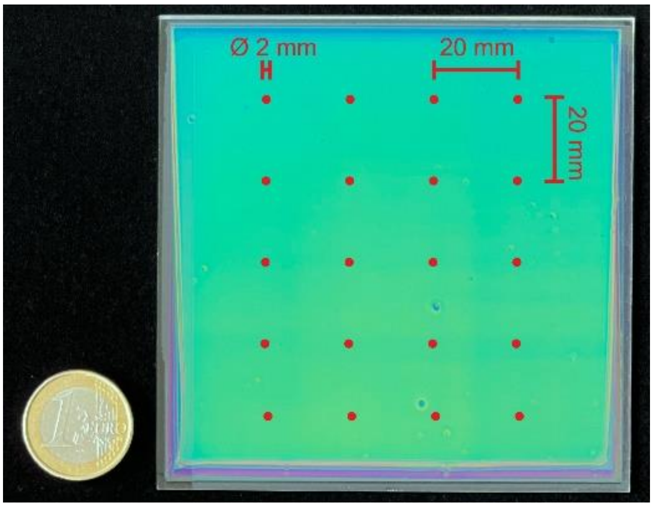

Supplementary Fig. 15. Sites for homogeneity characterization in 10 × 10 cm^2 filter.

13.3. Spectral values of the measured sites

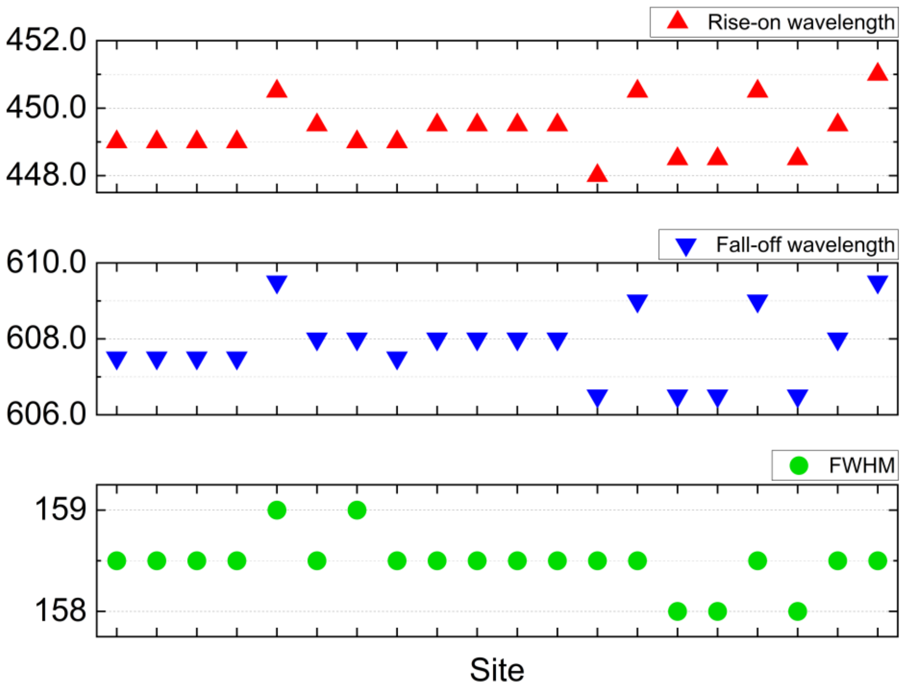

Supplementary Fig. 16. Characterization of the optical homogeneity. Rise-on (red), fall-off (blue) wavelengths, and FWHM (green) in 10 × 10 cm^2 filter.

14. A4 size (29.7 × 21.0 cm^2) dichroic filter

To characterize the homogeneity of the optical property in the printed A4 filter, an array consisting of 20 sites was used. Optical transmission measurements were performed at each site.

The size of the light spot was 2 mm. The horizontal and vertical distances between the neighboring sites are 35 and 40 mm, respectively.

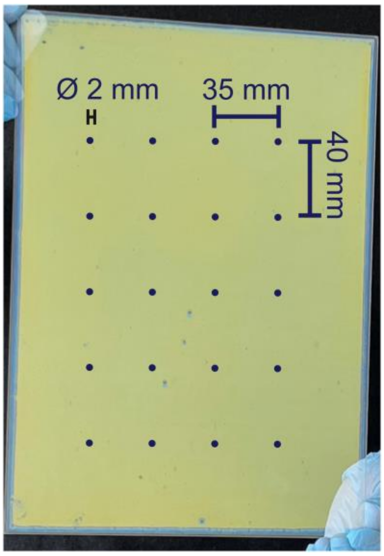

Supplementary Fig. 17. Sites for homogeneity characterization in A4 size dichroic filter.

15. Printing OIFs on flexible foil

To show the possibility of applying the developed printing process on a flexible substrate. Patterned OIFs were designed and printed on PET foil (Puetz Folien). The size of the foil substrate is 9 × 9 cm².

The parameters in the printing remained the same. The changed parameters are the annealing temperature and the time. The annealing temperature was reduced from 200 °C to 100 °C, and the annealing time increased from 10 min to 1 hour for each printed single layer. As a proof of concept, the total layer number of the printed OIFs is 8 in Supplementary Fig. 18.

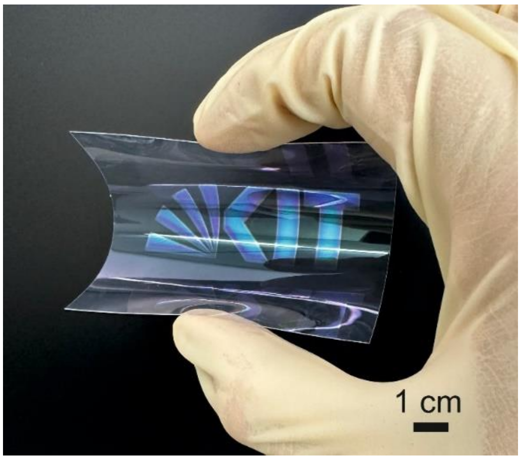

Supplementary Fig. 18. Patterned OIFs printed on a flexible PET foil.

16. Residual transmission elimination

In optical filter design, expanding the optical stopband is a common strategy to eliminate miscellaneous transmission. This can be realized by stacking multiple optical cavities. In this work, the printed longpass filter has a cut-on wavelength of 532 nm. To suppress the miscellaneous transmittance in the 400 to 450 nm wavelength range, a second cavity behaving as another longpass filter can be stacked on the original cavity.

Here, we show a proof of concept of suppressing the undesired transmission. Supplementary Fig. 19a shows the designed single cavity longpass filter in this work, namely the original cavity. Supplementary Fig. 19b shows the transmission curve of a second cavity. By stacking these two cavities, the residual transmission between 400 and 450 nm can be suppressed, as shown in Supplementary Fig. 19c. Furthermore, optimization of individual layer thickness and the total layer number of each optical cavity can be applied based on the new stack.

The strategy shown above can be used to eliminate undesired transmission in longpass, shortpass, and bandpass filters.

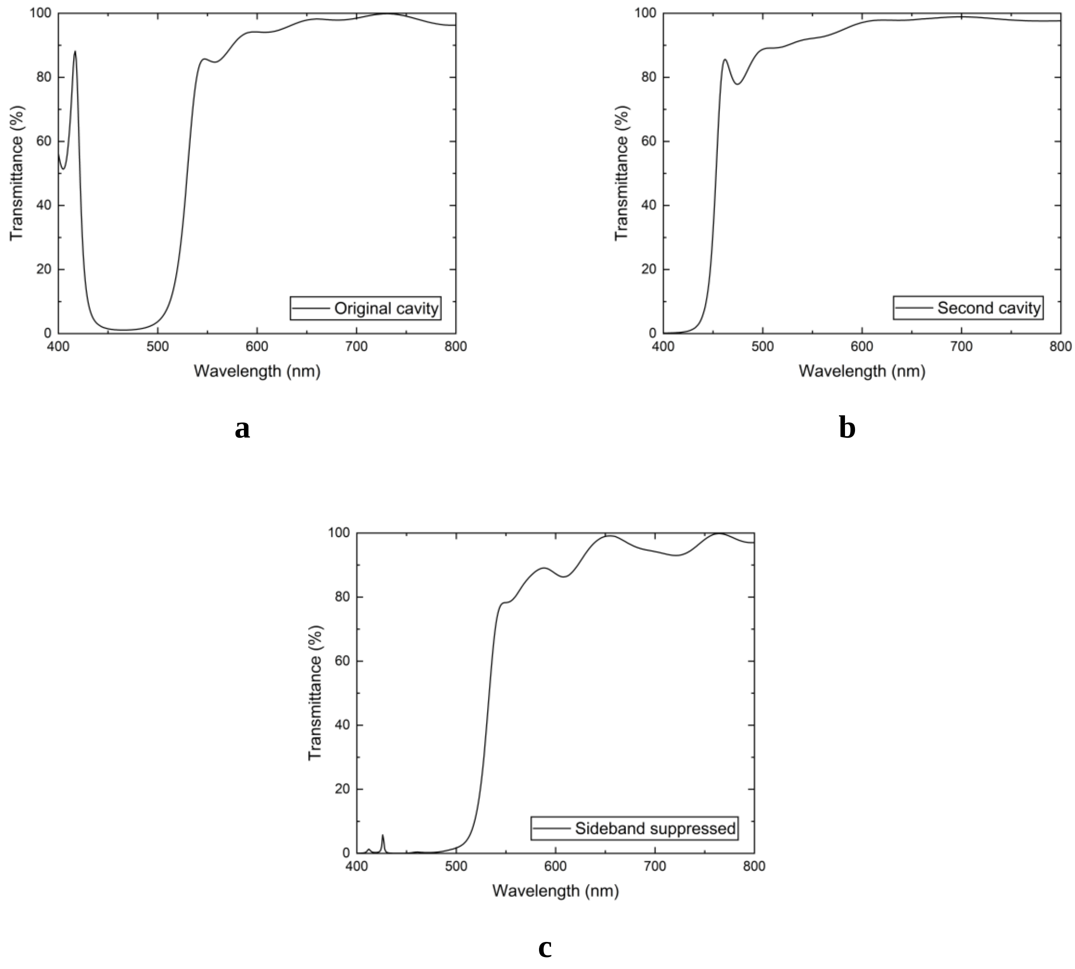

Supplementary Fig. 19. Transmittance of filters with different designs. **a** Original cavity. **b** Second cavity. **c** Sideband suppression by stacking the second cavity on the original cavity.

17. Comparison with commercial products

Here, we compare our inkjet-printed OIFs with the commercial products purchased from Edmund Optics. The filters compared are longpass (LP) and shortpass (SP) filters similar to our filters. However, the cut-on/off wavelengths of each compared set are not exactly the same.

The reference filters are purchased from Edmund Optics. The detailed information is listed in the following table.

Principle	Fabricating method	Filter type	Cut on/off wavelength	Size	Online store item number
Interference	Vacuum approach	LP	500 nm	6 × 6 mm^2	15234
		SP	450 nm	6 × 6 mm^2	15200

17.1. Comparison of transmission between commercial and inkjet-printed OIFs

Compared to the commercial LP filter, which is fabricated in vacuum, the inkjet-printed LP filter shows a similar transmission in the pass region above 500 nm. The blocking behavior in the region 430-500 nm is good (approx. 3%) but lower than the commercial ones due to the lower number of layers, which can be improved by further adding layers. Furthermore, the residual transmission in the shorter wavelength range (400-450 nm) can be suppressed according to individual applications as discussed below (see subsection “Increase the optical density in inkjet-printed OIFs”).

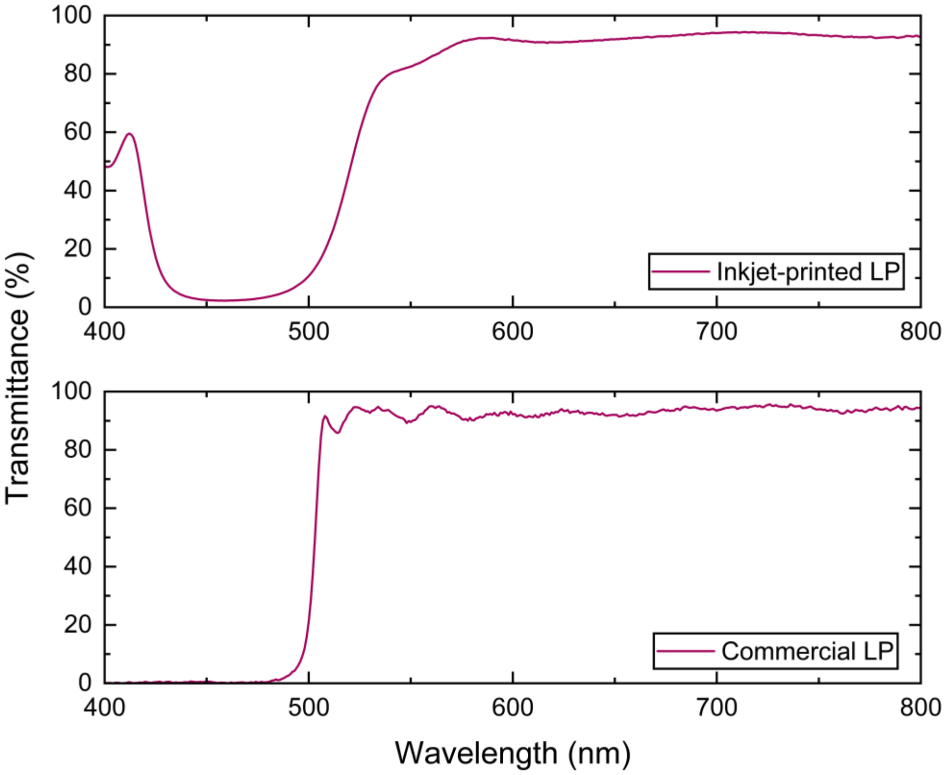

Supplementary Fig. 20. Comparison of longpass filters.

Compared to the commercial SP filter, which is fabricated in vacuum, the inkjet-printed SP filter shows an acceptable transmission in the pass region. The transmission in the pass region can be further smoothed by optimizing layer thicknesses. Both filters have a residual transmission in the longer wavelength range, which can be further suppressed according to individual applications by more complex layer stacks.

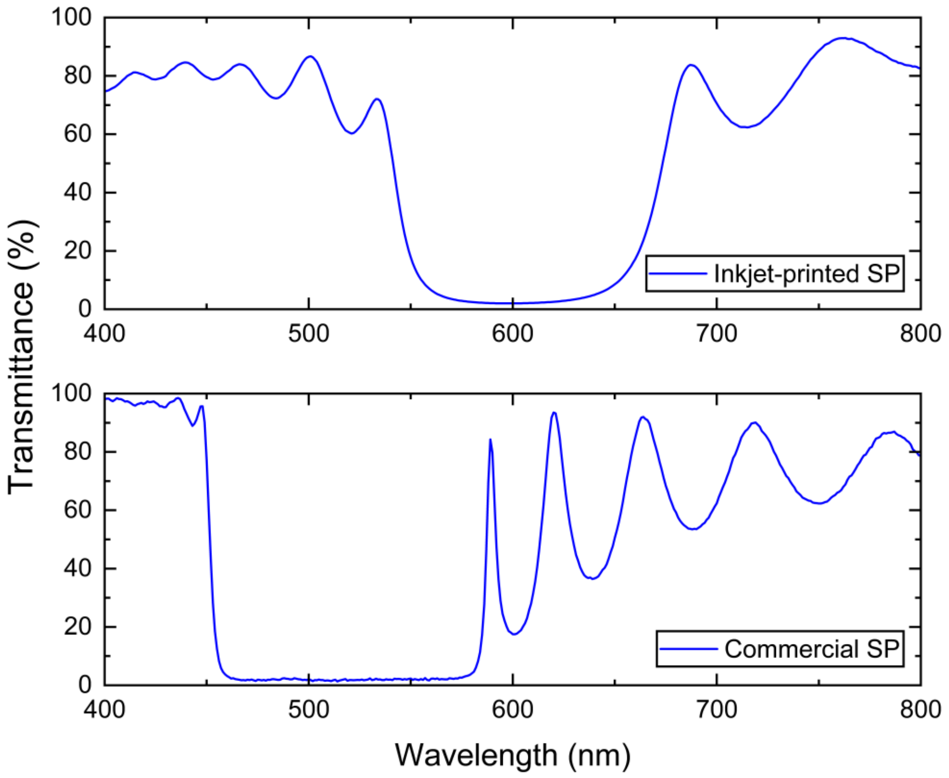

Supplementary Fig. 21. Comparison of shortpass filters.

17.2. Increase the optical density in inkjet-printed OIFs

It can be seen that the printed OIFs can be further optimized to achieve a higher optical density (OD) in the blocking range. To realize this, more layers can be added in future work. To better illustrate how to further improve the printed OIFs, we address the optical OD improvement as an example in this section. OD is calculated as

$$OD = -log_{10} T$$

where T is the transmittance of the filter. OD is used to describe how well the optical power can be attenuated or blocked. A higher OD means a better capability of rejecting the light in the spectral region of low transmission. For instance, Supplementary Fig. 22 shows an LP filter requiring high transmission above 450 nm and suppressing the light transmission below 450 nm. With an increased total layer number, the OD goes up to 9. Nowadays, a typical OD for a commercial optical interference filter is between 2 and 6.

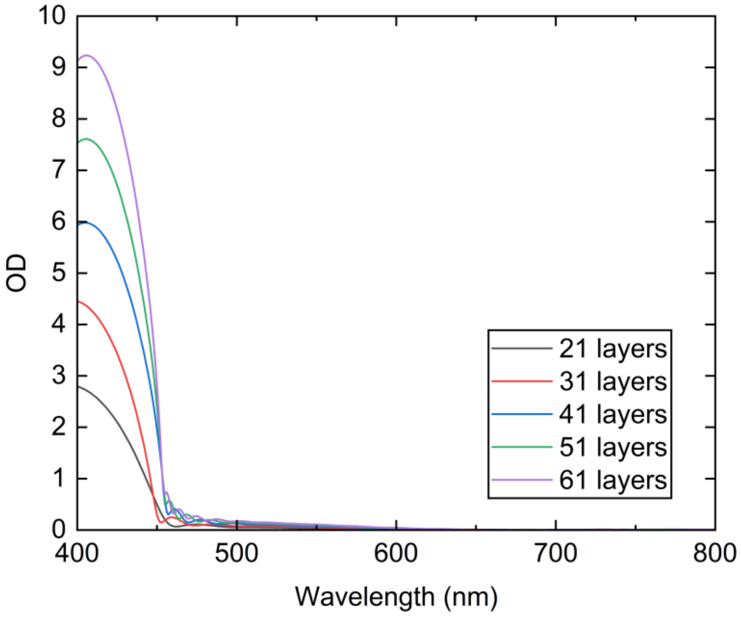

Supplementary Fig. 22. OD increases with layer number.

18. Durability test of printed layers

To characterize the mechanical performance of the printed filter. Adhesion and moderate abrasion tests were carried out based on the standards MIL-PRF-13830B and DIN EN ISO 2409.

- Adhesion characterization (MIL-PRF-13830B and DIN EN ISO 2409)

The testing samples were two filters consisting of 4 printed layers, which were annealed at 200 °C and 500 °C for 10 mins, respectively. The temperature-increasing slope was 200 °C per hour, and the samples were naturally cooled down to room temperature. Before performing the durability tests, the samples were placed in a test chamber with a temperature of 48 ± 3 °C and 95% to 100% relative humidity for 24 hours (according to MIL-PRF-13830B, C.4.5.8).

Each surface was crosscut by a steel cutter. Cellophane tape was firmly pressed against the surface and quickly removed at an angle that was normal to the surface. The results are presented in the Supplementary Table 3.

Supplementary Table 3. Adhesion test based on different annealing temperatures.

Annealing	Crosscut, before applying the tape	Crosscut, after applying the tape	Area removed	Classification
200 °C, 10 mins	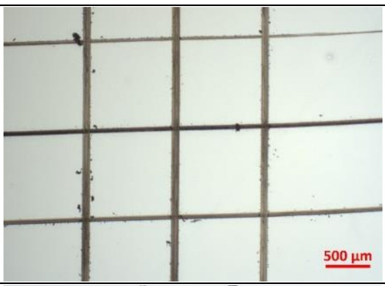	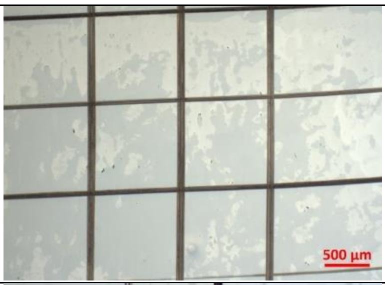	>65%	ISO Class 5
500 °C, 10 mins	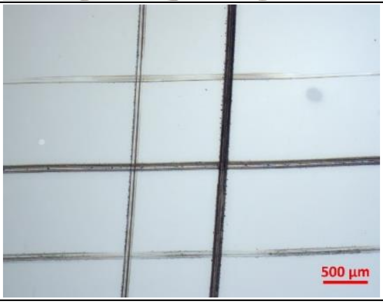	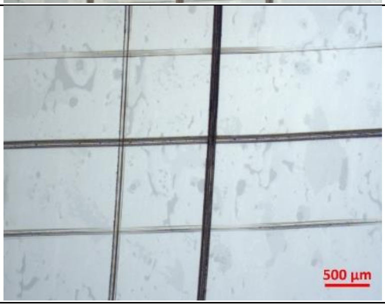	5-15%	ISO Class 2

According to the results, the adhesion performance can be improved by increasing the annealing temperature. Due to the limit of the used glass substrate, the annealing temperature was limited to 500 °C, and the filter adhesion classification is up to ISO Class 2.

Further increasing the adhesion between the nanoparticles and substrates, as well as the adhesion between the nanoparticles themselves, can be achieved by oligomers[26], including an interlayer[27], pre-treatment of substrate[28], modifying surface ligands of nanoparticles[29], and substrate surface roughness control[30].

- Moderate abrasion test (MIL-PRF-13830B, C.4.5.11)

Printed filters consisting of 4 layers were annealed at 200 °C for 24 hours. Followed by being placed in a test chamber with a temperature of 48 ± 3 °C and 95% to 100% relative humidity for 24 hours (according to MIL-PRF-13830B, C.4.5.8).

After this, the filters were rubbed with a pad of clean, dry, laundered cheesecloth (6.4 mm × 9.5 mm). The cheesecloth pad was rubbed across the surface from one point to another over the same path for 50 strokes with a force of 450 g continuously applied. The results are presented in the following Supplementary Fig. 23.

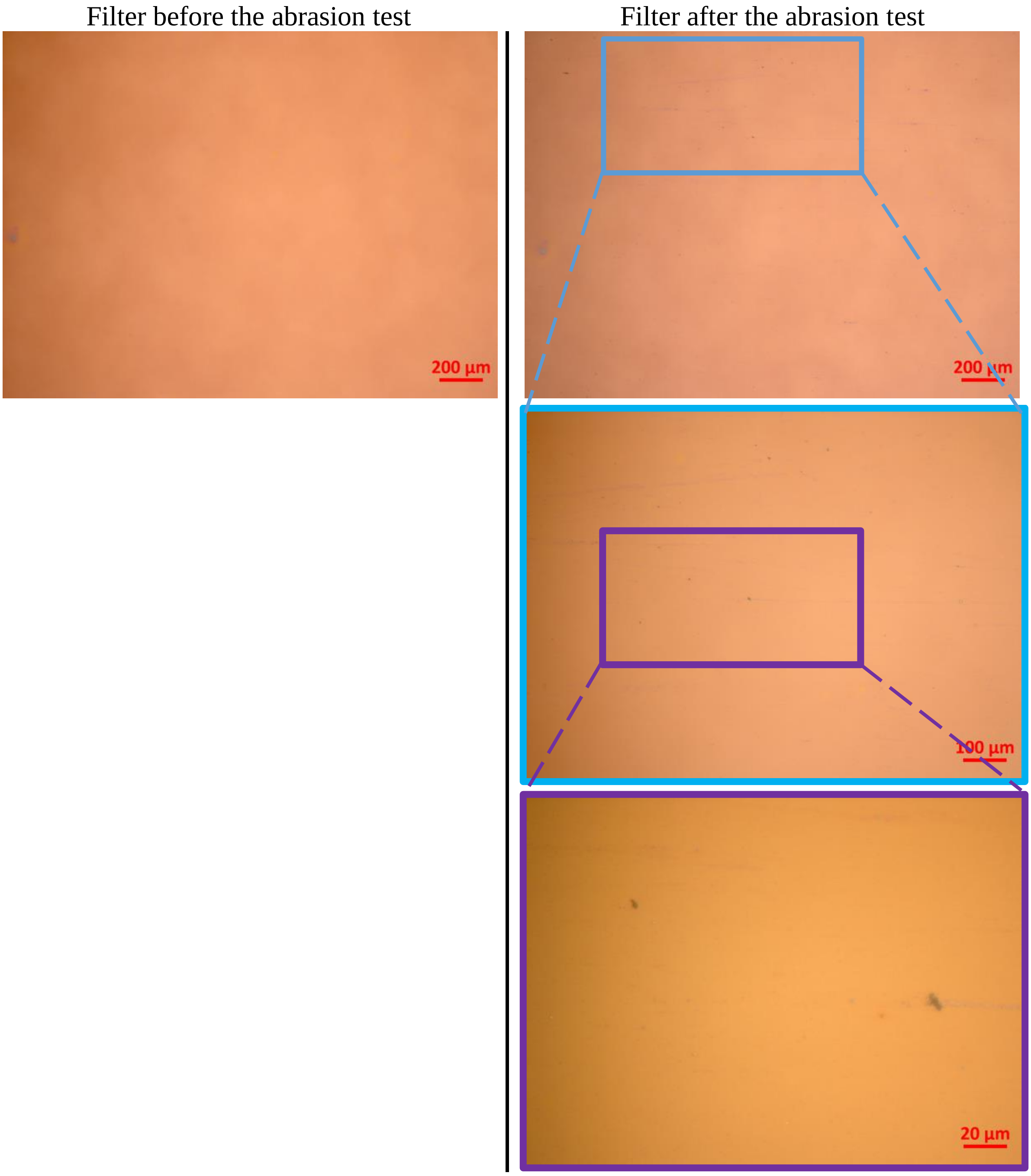

Supplementary Fig. 23. Optical images of printed filters before and after the abrasion test.

After the abrasion test, the filter did not show visible scratches according to the inspection method in Standard MIL-PRF-13830B. The induced defects can only be seen using a light microscope. The width of the scratch width is within 5 µm.

According to the standard MIL-PRF-13830B, the scratch number is used to classify the surface quality grade. The scratch number and the corresponding scratch width are in the following table.

Supplementary Table 4. Scratch number and the corresponding scratch width.

Scratch number	Scratch width (mm)
5	0.005
10	0.010
20	0.020
40	0.040
60	0.060
80	0.080
120	0.120

Based on these results, the surface quality of the printed layers is classified as Scratch number 5, denoted as the highest surface quality.

19. Crystal structure characterization of nanoparticles

X-ray diffraction analysis (XRD) was performed to characterize the crystallographic structure of the nanoparticles in the inks. The results show that the SiO_2 nanoparticles are in the amorphous phase[22,23]. The TiO_2 nanoparticles are in the rutile phase[24,25]. The annealing process did not change the phase of the nanoparticles.

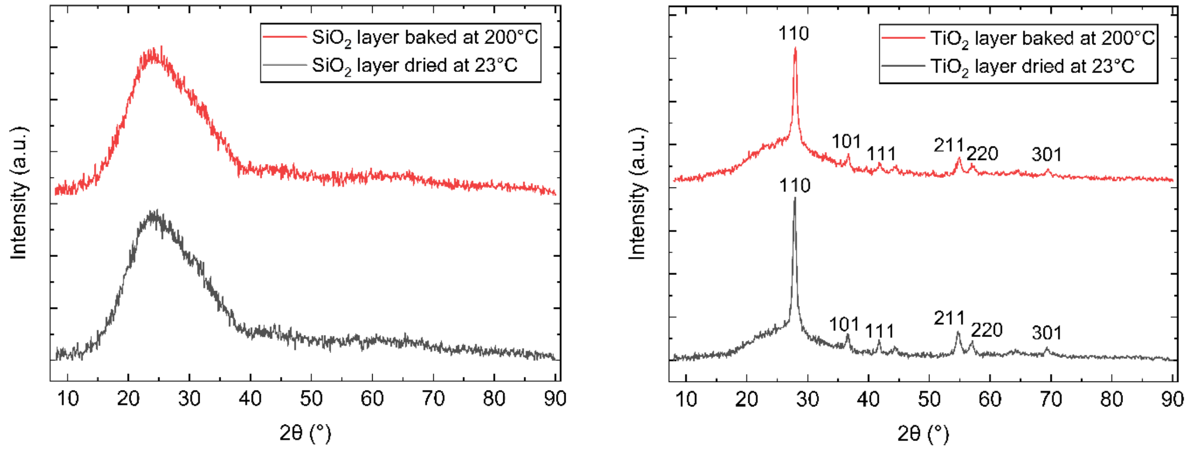

Supplementary Fig. 24. XRD patterns of nanoparticles.

20. Designed thickness of the filters

In this section, the designed thicknesses of optical filters are shown. The values are obtained from the simulation software Essential Macleod.

Supplementary Table 5. Designed thickness of the individual layers of the longpass, shortpass, and bandpass filters.

	Longpass filter		Shortpass filter		Bandpass filter	
Layer No.	Material	Thickness (nm)	Material	Thickness (nm)	Material	Thickness (nm)
1	SiO_2	153.20	TiO_2	78.71	SiO_2	153.20
2	TiO_2	69.57	SiO_2	119.05	TiO_2	69.57
3	SiO_2	70.12	TiO_2	76.93	SiO_2	70.12
4	TiO_2	52.02	SiO_2	116.96	TiO_2	52.02
5	SiO_2	114.15	TiO_2	75.94	SiO_2	114.15
6	TiO_2	47.35	SiO_2	117.23	TiO_2	47.35
7	SiO_2	87.63	TiO_2	78.62	SiO_2	87.63
8	TiO_2	61.70	SiO_2	110.40	TiO_2	61.70
9	SiO_2	87.63	TiO_2	78.62	SiO_2	87.63
10	TiO_2	61.70	SiO_2	110.40	TiO_2	61.70
11	SiO_2	87.63	TiO_2	78.62	SiO_2	87.63
12	TiO_2	61.70	SiO_2	114.09	TiO_2	61.70
13	SiO_2	70.13	TiO_2	80.89	SiO_2	70.13
14	TiO_2	72.29	SiO_2	110.02	TiO_2	72.29
15	SiO_2	92.04	TiO_2	81.61	SiO_2	92.04
16	TiO_2	45.06	SiO_2	112.45	TiO_2	45.06
17	SiO_2	82.13	TiO_2	83.49	SiO_2	82.13
18	TiO_2	76.34	SiO_2	120.24	TiO_2	76.34
19			TiO_2	83.48	SiO_2	49.31
20					SiO_2	118.06
21					TiO_2	78.71
22					SiO_2	119.05
23					TiO_2	76.93
24					SiO_2	116.96
25					TiO_2	75.94
26					SiO_2	117.23
27					TiO_2	78.62
28					SiO_2	110.40
29					TiO_2	78.62
30					SiO_2	110.40
31					TiO_2	78.62
32					SiO_2	78.40
33					TiO_2	114.09
34					SiO_2	80.89
35					TiO_2	110.02
36					SiO_2	81.61
37					TiO_2	83.45
38					SiO_2	120.24
39					TiO_2	83.48

Supplementary Table 6. Designed thickness of the individual layers of the bandpass filter based on a Fabry-Pérot etalon without a silver layer.

Layer No.	Material	Thickness (nm)
1	TiO_2	77.39
2	SiO_2	105.50
3	TiO_2	77.39
4	SiO_2	105.50
5	TiO_2	77.39
6	SiO_2	105.50
7	TiO_2	77.39
8	SiO_2	105.50
9	SiO_2	105.50
10	TiO_2	77.39
11	SiO_2	105.50
12	TiO_2	77.39
13	SiO_2	105.50
14	TiO_2	77.39
15	SiO_2	105.50
16	TiO_2	77.39

Supplementary Table 7. Designed thickness of the individual layers of the bandpass filter based on a Fabry-Pérot etalon with a silver layer.

Layer No.	Material	Thickness (nm)
1	TiO_2	77.39
2	SiO_2	105.50
3	TiO_2	77.39
4	SiO_2	105.50
5	TiO_2	77.39
6	SiO_2	105.50
7	TiO_2	77.39
8	SiO_2	105.50
9	SiO_2	70.00
10	Ag	65.00
11	SiO_2	70.00
12	SiO_2	105.50
13	TiO_2	77.39
14	SiO_2	105.50
15	TiO_2	77.39
16	SiO_2	105.50
17	TiO_2	77.39
18	SiO_2	105.50
19	TiO_2	77.39

Supplementary Table 8. Designed thickness of the individual layers of dichroic filters with different center wavelength (CWL).

		CWL380	CWL480	CWL590	CWL620	CWL680
Layer No.	Material	Thickness (nm)	Thickness (nm)	Thickness (nm)	Thickness (nm)	Thickness (nm)
1	TiO2	48.59	65.59	83.21	86.98	94.75
2	SiO2	69.20	86.20	107.15	111.89	121.72
3	TiO2	48.59	65.59	83.21	86.98	94.75
4	SiO2	69.20	86.20	107.15	111.89	121.72
5	TiO2	48.59	65.59	83.21	86.98	94.75
6	SiO2	69.20	86.20	107.15	111.89	121.72
7	TiO2	48.59	65.59	83.21	86.98	94.75
8	SiO2	69.20	86.20	107.15	111.89	121.72
9	TiO2	48.59	65.59	83.21	86.98	94.75
10	SiO2	69.20	86.20	107.15	111.89	121.72
11	TiO2	48.59	65.59	83.21	86.98	94.75
12	SiO2	69.20	86.20	107.15	111.89	121.72
13	TiO2	48.59	65.59	83.21	86.98	94.75
14	SiO2	69.20	86.20	107.15	111.89	121.72
15	TiO2	48.59	65.59	83.21	86.98	94.75
16	SiO2	69.20	86.20	107.15	111.89	121.72
17	TiO2	48.59	65.59	83.21	86.98	94.75
18	SiO2	69.20	86.20	107.15	111.89	121.72

21. Transmittance of glass substrate

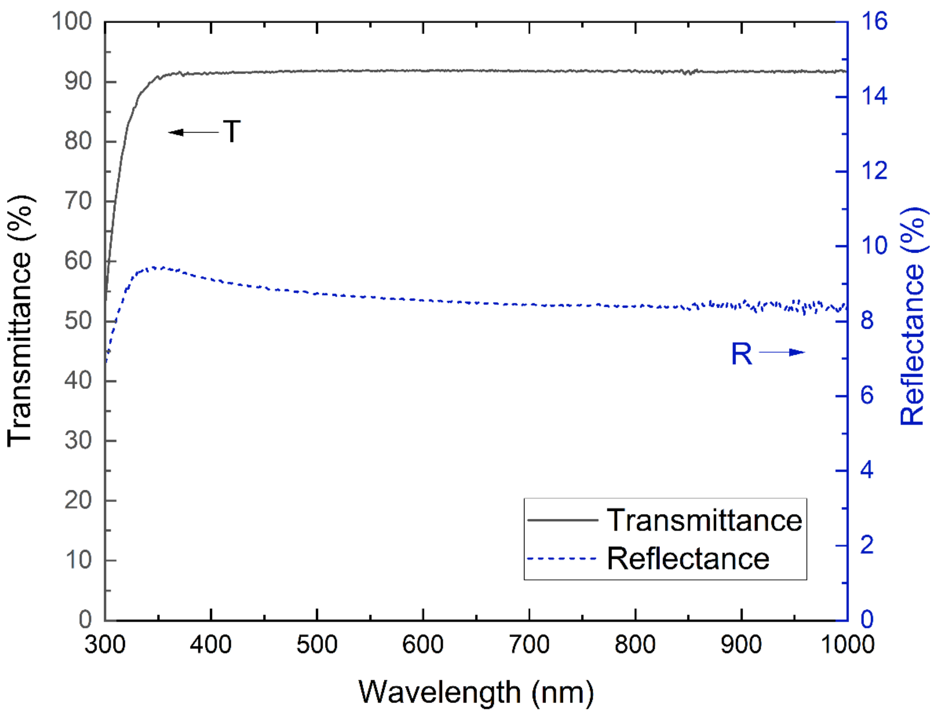

Supplementary Fig. 25. Transmittance and reflectance curve of glass substrate.

Supplementary References

1. Asghar, M. H., Shoaib, M., Placido, F. & Naseem, S. Modeling and preparation of practical optical filters. *Current Applied Physics* **9,** 1046–1053; 10.1016/j.cap.2008.11.007 (2009).
2. Martinu, L. & Poitras, D. Plasma deposition of optical films and coatings: A review. *Journal of Vacuum Science & Technology A: Vacuum, Surfaces, and Films* **18,** 2619–2645; 10.1116/1.1314395 (2000).
3. *Photonic Crystal and Its Applications for Next Generation Systems.* 1st ed. (Springer Nature Singapore, Singapore, 2023).
4. Musgraves, J. D., Hu, J. & Calvez, L. *Springer handbook of glass* (Springer, Cham, Switzerland, 2019).
5. *LICHT 2016. Karlsruhe, 25. - 28. September ; Tagungsband - Proceedings ; [22. Gemeinschaftstagung = 22nd Associations' Meeting]* (KIT Scientific Publishing, Karlsruhe, 2016).
6. Stone, M. C. *A field guide to digital color* (A K Peters/CRC Press, Place of publication not identified, 2016).
7. Baumeister, P. *Optical coating technology* (SPIE Optical Engineering Press, Bellingham WA., 2004).
8. Karim, M. A. *Electro-optical displays.* 1st ed. (CRC Press, Boca Raton, 2020).
9. Zapka, W. & Zapka, W. e. *Handbook of industrial inkjet printing. A full system approach / Werner Zapka.* 1st ed. (Wiley-VCH, Weinheim, 2017).
10. Träger, F. *Springer handbook of lasers and optics.* 2nd ed. (Springer, Dordrecht, New York, 2012).
11. Macleod, H. A. *Thin-film optical filters.* 4th ed. (CRC; London : Taylor & Francis [distributor], Boca Raton, Fla., 2010).
12. Young, T. III. An essay on the cohesion of fluids. *Phil. Trans. R. Soc.* **95,** 65–87; 10.1098/rstl.1805.0005 (1805).
13. Owens, D. K. & Wendt, R. C. Estimation of the surface free energy of polymers. *J. Appl. Polym. Sci.* **13,** 1741–1747; 10.1002/app.1969.070130815 (1969).

14. Verkuijlen, R., van Dongen, M., Stevens, A., van Geldrop, J. & Bernards, J. Surface modification of polycarbonate and polyethylene naphtalate foils by UV-ozone treatment and μPlasma printing. *Applied Surface Science* **290,** 381–387; 10.1016/j.apsusc.2013.11.089 (2014).

15. Cano-Raya, C., Denchev, Z. Z., Cruz, S. F. & Viana, J. C. Chemistry of solid metal-based inks and pastes for printed electronics – A review. *Applied Materials Today* **15,** 416–430; 10.1016/j.apmt.2019.02.012 (2019).

16. Liu, Q. *et al.* Enhanced pseudo-piezoelectric dynamic force sensors based on inkjet-printed electrostrictive terpolymer. *Organic Electronics* **67,** 259–271; 10.1016/j.orgel.2019.01.028 (2019).

17. Liu, Y. & Derby, B. Experimental study of the parameters for stable drop-on-demand inkjet performance. *Physics of Fluids* **31,** 32004; 10.1063/1.5085868 (2019).

18. Hoath, S. D. (ed.). *Fundamentals of inkjet printing. The science of inkjet and droplets* (Wiley-VCH Verlag, Weinheim, Germany, 2016).

19. Zhang, Q. *et al.* Fabrication of Bragg Mirrors by Multilayer Inkjet Printing. *Advanced materials (Deerfield Beach, Fla.)* **34,** e2201348; 10.1002/adma.202201348 (2022).

20. Liu, Y.-F., Hwang, W.-S., Pai, Y.-F. & Tsai, M.-H. Low temperature fabricated conductive lines on flexible substrate by inkjet printing. *Microelectronics Reliability* **52,** 391–397; 10.1016/j.microrel.2011.05.007 (2012).

21. Rodriguez-Rivero, C., Castrejón-Pita, J. R. & Hutchings, I. M. Aerodynamic Effects in Industrial Inkjet Printing. *jist* **59,** 40401-1-40401-10; 10.2352/J.ImagingSci.Technol.2015.59.4.040401 (2015).

22. Nayak, P. P. & Datta, A. K. Synthesis of SiO2-Nanoparticles from Rice Husk Ash and its Comparison with Commercial Amorphous Silica through Material Characterization. *Silicon* **13,** 1209–1214; 10.1007/s12633-020-00509-y (2021).

23. Sompech, S., Dasri, T. & Thaomola, S. Preparation and Characterization of Amorphous Silica and Calcium Oxide from Agricultural Wastes. *Orient. J. Chem* **32,** 1923–1928; 10.13005/ojc/320418 (2016).

24. Mayabadi, A. H. *et al.* Evolution of structural and optical properties of rutile TiO2 thin films synthesized at room temperature by chemical bath deposition method. *Journal of Physics and Chemistry of Solids* **75,** 182–187; 10.1016/j.jpcs.2013.09.008 (2014).

25. You, Y. F. *et al.* Structural characterization and optical property of TiO2 powders prepared by the sol–gel method. *Ceramics International* **40,** 8659–8666; 10.1016/j.ceramint.2014.01.083 (2014).

26. Zhao, X. & Murphy, M. C. A High-adhesion Binding Strategy for Silica Nanoparticle-based Superhydrophobic Coatings. *Colloids and surfaces. A, Physicochemical and engineering aspects* **625**; 10.1016/j.colsurfa.2021.126810 (2021).

27. Zhu, R. *et al.* Improved adhesion of interconnected TiO2 nanofiber network on conductive substrate and its application in polymer photovoltaic devices. *Applied physics letters* **93**; 10.1063/1.2907317 (2008).

28. Yin, Y. *et al.* Improving Adhesion Between Nanoparticles and Surface of Mica Substrate by Aminosilane Modification. *Plasmonics* **15,** 399–407; 10.1007/s11468-019-01030-8 (2020).

29. Sekhavat Pour, Z., Ghaemy, M., Bordbar, S. & Karimi-Maleh, H. Effects of surface treatment of TiO2 nanoparticles on the adhesion and anticorrosion properties of the epoxy coating on mild steel using electrochemical technique. *Progress in Organic Coatings* **119,** 99–108; 10.1016/j.porgcoat.2018.02.019 (2018).

30. Cedillo-Gonzalez, E. I., Montorsi, M., Mugoni, C., Montorsi, M. & Siligardi, C. Improvement of the Adhesion Between TiO2 Nanofilm and Glass Substrate by Roughness Modifications. *Physics Procedia* **40,** 19–29; 10.1016/j.phpro.2012.12.003 (2013).
